# A thigh-stakes diagnosis: Recurrent acral melanoma presenting as a rapidly enlarging mass

**DOI:** 10.1016/j.jdcr.2026.03.040

**Published:** 2026-03-27

**Authors:** Erik H. Pham, Leo P. Wu, Grant M. Pham

**Affiliations:** a Texas A&M University, College Station, Texas; b Pathology Department, Endeavor Health Elmhurst Hospital, Elmhurst, Illinois; c Family Dermatology, Arlington, Texas

**Keywords:** acral lentiginous melanoma, dermatopathology, immunohistochemistry, skin of color, soft tissue metastasis

## Introduction

Melanoma is an aggressive malignancy of melanocytes that accounts for approximately 8500 deaths annually in the United States.^1^ The prevalence of cutaneous melanoma variants differs across demographic groups. One rare but clinically significant subtype is acral lentiginous melanoma (ALM), which arises on the palms, soles, and subungual regions. As described by Shannon et al., ALM accounts for approximately 2% to 3% of all melanoma diagnoses in the general population but constitutes a disproportionately higher fraction of cases among individuals with darker skin phototypes, up to 33% to 36%.^2^ Despite its lower overall incidence, ALM often presents at later stages and has poorer prognosis due to delayed recognition and greater depth of invasion upon diagnosis.^3^

While cutaneous spread, lymphatic dissemination, and visceral metastases are well described in melanoma, subcutaneous recurrences in soft tissues, particularly at noncontiguous sites, can mimic other neoplasms and pose significant diagnostic challenges.^4^ In patients with a history of ALM, including prior toe or foot lesions, recurrence in distant soft tissue locations such as the proximal thigh may represent in-transit metastasis, regional nodal basin extension, or distant subcutaneous metastasis.^5^ Histopathologic examination is essential to differentiate recurrent melanoma from other soft tissue tumors, including rare melanocytic sarcomas such as clear cell sarcoma (CCS).^6^^,^^7^ To our knowledge, there are limited reports of acral lentiginous melanoma recurring as a large, deep, noncontiguous soft tissue mass without epidermal involvement. We report this unusual presentation to highlight the diagnostic value of a morphology-first approach supported by targeted immunohistochemical and molecular studies.

## Case presentation

**Fig 1:**
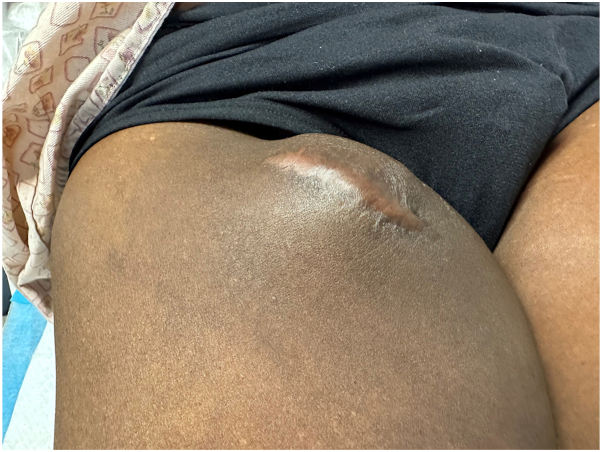
Acral melanoma recurrence presenting as a subcutaneous mass in the right proximal thigh. The mass is firm and well-circumscribed beneath intact overlying skin. No epidermal change or ulceration is evident.

A 66-year-old African American woman with a history of right-foot acral melanoma treated by digital amputation 2 years prior presented with a rapidly enlarging mass in the right proximal thigh. The stage of the original tumor was unknown because the procedure and diagnostic evaluation were performed in Kenya and records were unavailable. Six months before presentation, an epidermal inclusion cyst (EIC) was excised from the same region. Approximately 3 months later, she noted progressive enlargement of a new mass at the site. Examination revealed a firm subcutaneous mass measuring 6.4 × 4.7 × 4.5 cm without overlying epidermal change. Excisional biopsy demonstrated malignant melanoma on hematoxylin and eosin stain (H&E), with immunohistochemistry (IHC) positive for SOX10, Melan-A, and HMB-45. Fluorescence in situ hybridization (FISH) was negative for EWSR1 rearrangement, excluding CCS. The patient was promptly referred to medical oncology for staging and melanoma-directed therapy (Fig 1).

## Results

### Histopathologic findings

**Fig 2:**
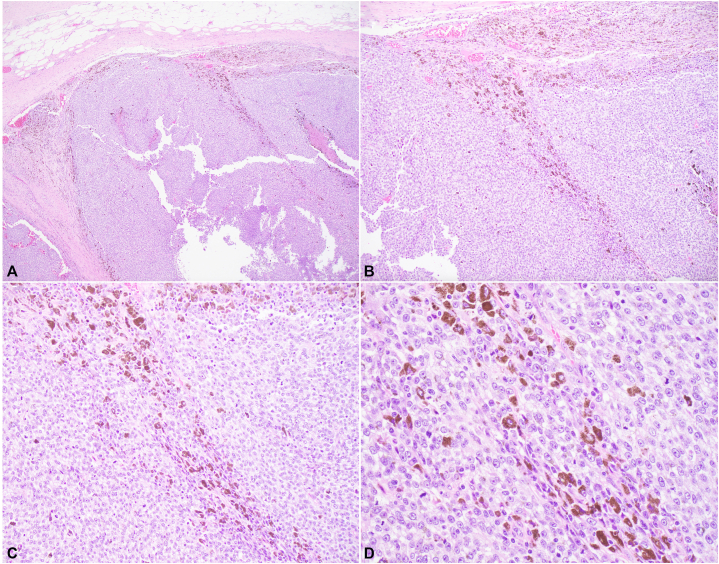
Histopathology of the thigh mass using hematoxylin and eosin (H&E) stain. **A,** Low-power (4×) view showing nodular-to-nested growth in the deep dermis and subcutis without epidermal involvement, consistent with dermal recurrence. **B** and **C,** Intermediate-power (10×-20×) views demonstrating architectural asymmetry, coalescing nests of atypical melanocytes, and coarse brown pigmentation. **D,** High-power (40×) view revealing epithelioid cells with pleomorphic nuclei, prominent nucleoli, abundant melanin-containing cytoplasm, and brisk mitoses.

At low magnification (Fig 2, *A*, 4×), the lesion involved the deep dermis and subcutaneous tissue and demonstrated a nodular-to-nested growth pattern with infiltrative extension into surrounding adipose tissue. No epidermal or junctional melanocytic component was identified, favoring a metastatic rather than primary cutaneous process.

At intermediate power (Fig 2, *B* and *C*, 10×-20×), the tumor showed architectural asymmetry, poor circumscription, and coalescing nests and sheets of atypical melanocytes. Regions of coarse brown pigmentation were present.

At high power (Fig 2, *D*, 40×), the neoplastic cells were predominantly epithelioid with enlarged pleomorphic nuclei, irregular nuclear membranes, prominent nucleoli, and abundant pale-to-eosinophilic cytoplasm containing coarse melanin pigment. Brisk mitotic activity, including deep and atypical mitoses, was readily identified.

### Immunohistochemistry (corroborative)

**Fig 3:**
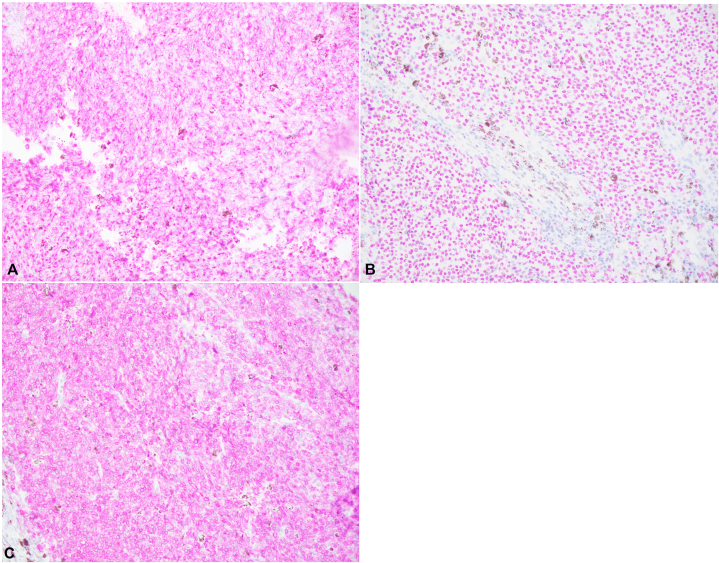
Immunohistochemical staining of the recurrent acral melanoma lesion (20×). The tumor demonstrates diffuse nuclear positivity for SOX10 **(B)** and diffuse cytoplasmic positivity for Melan-A **(C)** and HMB-45 **(A)**, confirming melanocytic differentiation and supporting a metastatic phenotype.

The tumor showed diffuse, strong nuclear positivity for SOX10 (Fig 3, *B*) and diffuse, strong cytoplasmic staining for Melan-A (Fig 3, *C*) and HMB-45 (Fig 3, *A*), confirming melanocytic differentiation. Staining was uniform throughout the lesion without evidence of maturation.

### Molecular analysis (ancillary)

FISH for EWSR1 rearrangement was negative, excluding CCS from the differential diagnosis.

## Discussion

On H&E, melanoma exhibits a broad morphologic spectrum but is classically characterized by architectural asymmetry, poor circumscription, brisk mitotic activity, lack of maturation with depth, and marked cytologic atypia, including nuclear pleomorphism and prominent eosinophilic nucleoli, often with melanin pigmentation.^8^^,^^9^ In this case, these morphologic features were readily apparent, supporting the diagnosis of metastatic melanoma; furthermore, the lesion’s deep soft tissue location and absence of epidermal and junctional involvement favor a metastatic process rather than a primary cutaneous melanoma. The lesion was also clearly distinct from a recurrent epidermal inclusion cyst (EIC), which was suspected clinically; EICs are typically well-circumscribed, cystic, lined by stratified squamous epithelium, and lack cytologic atypia or mitotic activity.

The histomorphologic findings in this case were highly characteristic of malignant metastatic melanoma; immunohistochemistry was subsequently performed to confirm melanocytic lineage. Similar observations have been reported by Arima et al., in which in-transit metastases from acral melanoma were diagnosed primarily on morphologic grounds, with IHC used for confirmation.^10^ This approach is particularly relevant in patients with a known history of melanoma, in whom morphology can guide selective ancillary testing while avoiding unnecessary diagnostic delay.

### Differential considerations

The primary clinical consideration in this case was recurrent or metastatic melanoma versus a benign soft tissue process, particularly given the history of EIC excision at the same site. Although a recurrent EIC was initially considered, intraoperative gross findings were not typical of a benign cystic lesion. Histologic evaluation demonstrated absence of cystic architecture with cytologic atypia and mitotic activity on H&E, excluding a benign cystic process. Final histopathology confirmed metastatic melanoma (Table I).

**Table I:** Case differential considerations for deep soft tissue mass in a patient with prior acral melanoma

Category	In-transit metastasis (acral melanoma)	Epidermal inclusion cyst (EIC)	Clear cell sarcoma (CCS)
Location/pattern	Cutaneous or subcutaneous deposits between the primary acral melanoma and regional lymph nodes; often multiple nodules along lymphatic channels	Subcutaneous; often near prior trauma or surgery; usually solitary; superficial to deep dermis; well-circumscribed	Deep soft tissues of tendons, aponeuroses, often lower extremities (foot/ankle); can mimic melanoma
Clinical features	Firm dermal/subcutaneous nodules; usually multiple; may be pigmented; months–years after primary; sometimes ulcerate; lymphatic obstruction common	Firm or fluctuant; slow-growing; generally painless unless inflamed; no pigmentation; often mobile; overlying punctum may be present	Slow-growing mass; usually solitary; often painful; typically in young adults; may show pigmentation; can mimic melanoma clinically
Histopathology	Dermal/subcutaneous nests or sheets of melanoma cells; cytologic atypia; brisk mitoses; epidermotropism usually absent; may show melanosis	Cyst lined by stratified squamous epithelium with granular layer; central lamellated keratin; no cytologic atypia or mitoses; well-circumscribed; may show inflammatory reaction if ruptured	Nests, fascicles of clear to pale eosinophilic spindle or polygonal cells; vesicular nuclei; prominent nucleoli; frequent melanin granules; dense fibrous septa; zellballen-like architecture; mitoses less frequent than melanoma
IHC markers	Positive: S100, SOX10, Melan-A, HMB-45	Usually negative for melanocytic markers (S100, SOX10, Melan-A, HMB-45)	Positive: S100, SOX10, Melan-A (sometimes); HMB-45 variable; Negative for cytokeratins; MITF positive; often less diffuse HMB-45 than melanoma
Molecular findings	Typically BRAF V600E (less common in acral), NRAS, or KIT mutations; acral melanoma often shows KIT amplification, structural rearrangements	None; benign lesion; no oncogenic mutations	EWSR1–ATF1 fusion (most common); no BRAF/NRAS/KIT mutations
Diagnostic factors	History of acral primary; location between primary and node; multiple dermal nodules; melanoma IHC and molecular concordance with primary	Well-circumscribed cystic lesion; keratin-filled lumen; lack of cytologic atypia; correlation with prior trauma or cyst excision	Identification of EWSR1 fusion; S100/SOX10 positivity but melanoma driver mutations absent; deep soft tissue origin; cytomorphology distinct
Prognosis	Indicates regional disease progression; intermediate prognosis; risk of further regional/distant spread; outcome depends on burden and therapy	Benign; excellent prognosis; recurrence possible if incompletely excised	Generally poor; high local recurrence; significant risk for metastatic spread (lung, LN); prognosis worse with larger tumors or positive margins
Treatment	Surgical excision of nodules; isolated limb perfusion/infusion or intralesional therapy for unresectable lesions; immunotherapy (PD-1 ± CTLA-4); radiation for palliation	Simple excision; drainage if inflamed; no systemic therapy required	Wide excision with negative margins; adjuvant radiation for high-risk or margin-positive tumors

### Clinical and prognostic implications

The size, depth, noncontiguous location, and solitary nature of this lesion are atypical for acral melanoma recurrence. Most recurrences present as multiple small, superficial nodules near the primary tumor site or along regional lymphatic drainage pathways, often with dermal or epidermal involvement. In our patient, the tumor presented as a single, large, deep subcutaneous mass in the proximal thigh that clinically mimicked a benign cystic process lacking an epidermal component. Such uncommon characteristics increase the risk of misdiagnosis as a primary soft tissue neoplasm, highlighting the need for diagnostic vigilance and a morphology-first approach. Management follows standard protocols for regionally or distantly recurrent melanoma, including staging evaluation, surgical excision when feasible, and systemic therapy as indicated.

## Conclusion

Acral lentiginous melanoma may recur as a deep, solitary soft tissue mass at a distant, noncontiguous site and mimic benign or primary soft tissue neoplasms. Recognition of classic histopathologic features on H&E, supported by focused ancillary testing, can expedite accurate diagnosis and appropriate oncologic management.

## Conflicts of interest

None disclosed.
